# Bringing Sexual and Reproductive Health in the Urban Contexts to the Forefront of the Development Agenda: The Case for Prioritizing the Urban Poor

**DOI:** 10.1007/s10995-013-1414-7

**Published:** 2013-12-19

**Authors:** Blessing Mberu, Joyce Mumah, Caroline Kabiru, Jessica Brinton

**Affiliations:** African Population and Health Research Centre, APHRC Campus, Manga Close, Off Kirawa Road, Kitisuru, P. O. Box 10787, Nairobi, 00100 Kenya

**Keywords:** Sexual and reproductive health, Maternal health, Urbanization, Policy, Program, Research

## Abstract

Estimates suggest that over 90 % of population increase in the least developed countries over the next four decades will occur in urban areas. These increases will be driven both by natural population growth and rural–urban migration. Moreover, despite its status as the world’s least urbanized region, the urban population in the sub-Saharan Africa region is projected to increase from under 40 % currently to over 60 % by 2050. Currently, approximately 70 % of all urban residents in the region live in slums or slum-like conditions. Sexual and reproductive health (SRH) risks for the urban poor are severe and include high rates of unwanted pregnancies, sexually transmitted infections, and poor maternal and child health outcomes. However, the links between poverty, urbanization, and reproductive health priorities are still not a major focus in the broader development agenda. Building on theoretical and empirical data, we show that SRH in urban contexts is critical to the development of healthy productive urban populations and, ultimately, the improvement of quality of life. We posit that a strategic focus on the sexual and reproductive health of urban residents will enable developing country governments achieve international goals and national targets by reducing health risks among a large and rapidly growing segment of the population. To that end, we identify key research, policy and program recommendations and strategies required for bringing sexual and reproductive health in urban contexts to the forefront of the development agenda.

## Background

Population growth challenges underlie virtually all millennium development goals (MDGs) [1–5]. There is consensus that the MDGs are difficult or impossible to achieve with prevailing levels of population growth in the least developed countries and regions. Following the overwhelming evidence on the importance of population growth is the global consensus that promoting sexual and reproductive health (SRH) through voluntary family planning is critical for the achievement of development goals [3, 4]. Besides its ability to contribute to the achievement of more clearly related goals, such as poverty reduction, maternal and child survival, and women’s empowerment through alleviating pressures caused by over-reproduction, the promotion of family planning is also linked to the attainment of less obviously salient objectives, such as environmental stability and access to natural resources, which are inextricably linked to population stabilization [2].

Since the 1994 ICPD conference, various mechanisms for monitoring and action frameworks have been enacted by various national, international and regional bodies to attain the SRH goals developed by this seminal conference. Consequently many countries in sub-Saharan Africa (SSA) have developed reproductive health policies and provided frameworks to guide implementation of SRH programs [6]. However, the notion that SRH is just health sector argument prevents many SSA countries and its leadership from adequately allocating resources to fund SRH programs, including inconsistent FP commodity supplies, has hindered the full realization of their commitments. Overall, although there has been progress in the policy space surrounding SRH in SSA countries, the implementation of SRH programs, especially those impacting the most vulnerable groups, remains problematic.

The heightened calls around repositioning of population issues in development agendas, policies, and programs, particularly in the context of SSA in recent years [7] and the current shift of discourses in the development sectors towards the post-MDGs agenda, provides a window of opportunity to highlight the importance of focus of new development goals on SRH in urban contexts across the global south, particularly in the SSA region. This commentary summarizes key policy, programmatic, and research priorities identified from (1) discussions with population experts, doctoral students, and policy makers and (2) an extensive review of published and grey literature on population dynamics, urbanization, and sexual and reproductive health.

## Increasing Urbanization of Poverty and Dwindling Urban Advantage

The year 2007 marked the world’s transition from a primarily rural sphere to a largely urban domain. Despite its status as the world’s least urbanized region, SSA currently has the fastest rate of urban growth in the world at between 3 and 4 % per annum, with urban population in the region projected to increase from 37 % in 2011 to about 57 % by 2050 [8]. Unless there are remarkable shifts in governance and economic growth, a large proportion of the urban population in SSA will live in dire poverty. In the face of increasing urbanization of poverty, it is estimated that approximately 70 % of all urban residents in the region will be living in slums or in slum-like environments [9, 10].

The growth of slums, which are characterized by overcrowding, social and economic marginalization, poor environmental conditions, insecurity, and little or no basic social services, has been linked to appreciable deterioration of key urban health and social indicators and reversals in favor of rural areas [10–13]. In particular, the new face of urban poverty has been linked to adverse SRH outcomes for the urban poor such as high rates of unwanted pregnancies, higher fertility, sexually transmitted infections (STIs), and poor maternal and child health outcomes. Evidence from Nairobi, the capital city of Kenya, shows that the fertility rate in the city’s informal settlements was higher than the Nairobi average (4.7 vs. 2.6). Similarly, child and infant mortality as well as disease prevalence, unemployment and poverty rates are higher than the city average [14, 15]. Nairobi slum residents exhibit significant disadvantages with respect to living conditions, morbidity, access to health services including family planning services, mortality, sexual violence and risky sexual practices relative to other population sub-groups, including rural residents [15–19]. Living in urban slums has been found to limit women’s ability to control their fertility and implement their fertility preferences. For example, in Korogocho and Viwandani slums in Nairobi city, almost half (47 %) of the pregnancies are unintended. Also, for about a quarter (28 %) of postpartum months where the risk of another pregnancy is high, no contraceptive method was used by women in the two slums [20]. Moreover, maternal mortality rates in the urban slums are almost double the national average, with 706 maternal deaths to 100,000 live births estimated, compared to 488 per 100,000 live births for all of Kenya [15].

Studies in several countries have yielded similar results with indicators such as child mortality rates being higher among the urban poor than among the rural population in several countries [9]. In the slums of Ibadan, Western Nigeria, Adedimeji et al. [21] found that 65 % of 1,600 youth aged 15–24 reported unprotected sex in the 3 months prior to the study. Although the sexually-active respondents demonstrated basic knowledge of HIV/AIDS and high risk perception of infection, risky behavior was common and protective behavior was poor. About 48 % of 505 males and 12 % of 537 females had multiple partners. Similarly, 29 % of males and 38 % of females were engaged in transactional sex, and only 14 % of males and 5 % of females used any form of protection, resulting in the high rates of STIs reported by 27 % of males and 10 % of females. The level of risky behavior observed against a background of high risk perception, suggests significant levels of constraint to adopting protective behaviors, with implications for high rates of STIs and the spread of HIV/AIDS. These constraints are rooted in the pervasive poverty and socioeconomic disparities that prevent many in the urban slums from practicing protective behaviors [22].

Building on the backdrop of the foregoing discussion and in the context of the more recent definition of reproductive health as complete well-being in sex and reproduction, highlighting not only family planning, conception, and birth, but also the imbalances in decision making autonomy between men and women, the possibilities of coercion and violence in relationships and exposure to difficult health risks [23, 24], the reproductive health disadvantage of the urban poor has become a key challenge in SSA. Consequently, the importance of a renewed focus in addressing the SRH challenges faced by the urban poor cannot be overstated. In fact, the point is made that many of the worst human problems, including poor health, play out together in city slums. The positive implications of effective SRH programs for maternal and child survival, morbidity and mortality outcomes have been well documented [25]. Effective SRH programs have provided a win–win solution not only on the improved welfare of individual women and children but also on national economies and the physical environment [26, 27].

Today, as the development sectors lobby for their own piece of the post-MDGs pie, it is vitally important that the post-2015 global development agenda includes focus on SRH in urban contexts. Building on our wide consultations with experts across the global north and south and triangulation of theoretical and empirical literature, we highlight key sexual and reproductive health priority areas for policy, programs, and research.

## Sexual and Reproductive Health Policy, Program, and Research Priorities in the Urban Context

### Policy Priorities

To date SRH policies have failed to account for the unique needs of vulnerable urban populations, including slum residents, street children, the disabled, urban refugees, internally-displaced persons, and cross-border migrants due, in part, to the misconception that urban residents have greater access to services and commodities relative to rural dwellers. However, the lack of state-sponsored health facilities in most urban informal settlements and the marginalization of other vulnerable populations, means that these groups face substantial challenges in accessing key medical and SRH services. It is therefore vital that politically-backed and well-funded SRH policies that target vulnerable urban sub-groups are developed and implemented.

As an initial step, African governments should endorse and advocate for implementation of urban specific versions of internationally-agreed health protocols, such as the Abuja Declaration, which requires governments in Africa to commit 15 % of their budgets to health. Governments should also support health initiatives that move beyond reliance on state-supported health care systems and instead create community development strategies utilizing community health workers (CHWs) and community-based organizations as well as partnerships with private outlets within the urban informal settlements. Increased use of CHWs and broadening of their roles to include maternal and child health services especially in resource poor settings has the potential to improve access to health care, especially use of facilities for crucial services such as delivery [28], and reduce the overall burden of disease. Rwanda presents a good example as the use of more than 45,000 CHWs combined with strong leadership has seen significant impact in health outcomes, with a 60 % reduction in maternal mortality ratio observed, while under-5 mortality has declined by 70 % since 2000. In the words of the Rwandan President Paul Kagame “use of community health workers is something we have had experience with and we have seen *the* good results.” [29] Further policy models that promote successful private–public partnerships in health service delivery should be explored, adapted and adopted in the urban context.

Adequate and sustainable funding for all categories of cities (small, medium and mega cities) for essential SRH services should be an important policy priority in the region. Creating itemized budget lines for sexual and reproductive health commodities and services targeting specific groups is recommended as one way to ensure funds are used for their intended purposes. In lieu of urban specific protocols; countries may need to include earmarks or higher levels of budgetary funding relative to the needs of the urban poor.

### Program Priorities

Programs to uplift the health of the urban poor must adopt a comprehensive platform that addresses socio-environmental and economic conditions that heighten vulnerability to poor health in urban contexts. Such a holistic approach can enhance the quality of life, reduce negative impacts on the environment, improve the overall health of a population, and reduce the burden of investment in curative health and poverty alleviation [30]. Poverty, in particular, must be recognized as a key hindrance to sexual and reproductive health in urban poor communities. Economic empowerment programs should, therefore, be incorporated into urban programming. Economic empowerment programs envisioned relevant to the urban poor will include programs to keep slum children in schools up to secondary levels, micro-credits programs, vocational skills training, and employment creation/business start-up grants. The UK aid strategic vision on girls presents a good example of an intervention strategy that takes a holistic approach to addressing structural poverty and poor SRH outcomes, which include education, economic asset building, delay in first pregnancy and support for safe birth, and prevention of violence against girls and women [31]. Linking up of these pillars will have greater impact on young women. For instance, strategies that will keep women in school will have impacts on maternal health as they will have fewer and healthier children, while improving their economic opportunities. If well implemented, this strategy has the potential to address the SRH burden among the urban poor.

Additional concerns that governments and development partners should consider when designing or implementing SRH programs in urban contexts include special consideration of the needs of the most vulnerable populations within urban communities, including youth, street children, the disabled, refugees, internally-displaced persons, and cross-border migrants. SRH interventions should be tailored to specific subsets of urban populations since the population diversity of people living in the slums greatly impacts the success and uptake of SRH services. Greater focus on free or subsidized quality reproductive health services, with specific attention paid to the provision of low cost maternity services to women, is imperative. The specific SRH needs of aging populations should also be integrated into SRH programs in urban areas including, but not limited to, the provision of breast, cervical and prostate cancer screening. Also, specific SRH health services for urban-based adolescents should be provided through a variety of non-traditional outlets including outreach camps, private provision of health services and in school health services, as well as door-to-door services, will ensure that all vulnerable youth in urban areas are reached.

Urban communities are a diverse and complex network. Differences in ethnic identities, cultural practices, and classifications such as migrants versus multi-generational slum dwellers as well as the inclusion of extremely vulnerable groups like street children, are all important factors that must be taken into consideration when designing and implementing a sexual and reproductive health program in urban poor communities. Urban communities, through their representative organizations, should be involved in health needs assessments, as well as the design, implementation and evaluation of programs aimed at improving their health and wellbeing. Finally, innovative approaches that raise awareness of sexual and reproductive health services, such as using mobile technology, should be further developed and adopted particularly for the hardest-to-reach among urban populations, such as women and youth in urban slums.

### Research Priorities

Commendable efforts have been made in research on SRH in SSA, with the extensive history of FP and SRH generally well documented. However, a strategic focus on SRH in urban contexts is critical to the development of healthy productive urban populations and, ultimately, the improvement of the quality of life for the rapidly increasing number of urban residents across the global south. To achieve improvements in SRH indicators in urban spaces, country governments and development partners must gain a better understanding of the dynamics of urbanization, the growth of slums and urbanization of poverty in order to provide services and improve the lives of urban dwellers in the global south. Country governments and development partners need to support high quality research that provides relevant evidence for policy and action on SRH in urban contexts, particularly among vulnerable sub-populations. Research data generated should include more detailed information to define urban categories, such as slum and non-slum areas, and should be disaggregated by age, sex, and socio-economic measures to enable planners and policymakers identify intra-city and inter-group commonalities and differentials and develop targeted policies and interventions. It is worth noting that national data sets such as the Demographic and Health Surveys, which are often the primary source of representative data on SRH do not currently allow for this level of disaggregation. The research agenda on SRH in urban contexts also needs to include efforts to establish the cost-effectiveness and feasibility of SRH interventions, such as government subsidies for services and supplies. There is also the need to collect data on urban specific system issues such as access to health services, with an emphasis on family planning, maternity care, and HIV/AIDS testing and treatment. This becomes important as a means to decipher context-specific hindrances to service provision and utilization.

## Conclusion

A comprehensive strategy of SRH policy, programs and research focused on the urban poor apart from its direct implications for improved SRH outcomes among urban and peri-urban dwellers, has the potential to diffuse to rural areas to which most urban dwellers are linked [32, 33]. Addressing poor SRH outcomes among the urban poor is critical, particularly because urban slums provide an expansive population—a fact which holds immense potential for making a large and much-needed impact. For example, there is a large market for contraceptives among this population due to lower desired family size than rural areas and the high number of unwanted births, meaning that there is also an opportunity to have a significant impact in the short term. As a corollary, family planning on its own, as a single intervention will have minimal impacts on the health of women and children, especially that of the urban poor. Extant literature posits that the integrated provision of family planning and maternal and child health services has implications for not only poverty reduction in resource poor settings, but significant declines in the maternal and child mortality rates [34]. Integration of family planning and maternal and child health services in urban informal settings therefore holds a great potential for significant impacts on the health of the mother and child, quality of services received and a long term impact on the overall wellbeing of families and their respective communities [35]. Finally, the significant role of poverty in the adverse SRH outcomes of the urban poor cannot be overlooked in policies and programs. To this effect, we argue that the SRH of the urban poor needs to be looked at both from a development perspective, as well as from a health sector and service delivery perspective. The need for multipronged approaches that include behavior change, job and wealth creation remains essential to addressing the SRH needs of the urban poor. Similarly, research efforts linking all these pillars, and documenting impacts, what works and identifying new and innovative approaches will be an important part of the process.
